# Enhancing penile function: the impact of a regenerative multimodal protocol on erectile dysfunction

**DOI:** 10.3389/frph.2025.1601354

**Published:** 2025-09-29

**Authors:** Andrés Soto-Rodríguez, Carla Pastora-Sesín, Juan Antonio Valverde-Espinoza, Sergio Campos-Sanchez, Massimiliano Mauro-Stamati, Vincent Giampapa, Víctor Urzola, José Rafael Rojas-Solano

**Affiliations:** ^1^ Research Unit, The Regenerative Medicine Institute, San José, Costa Rica; ^2^ Clinical Department, The Regenerative Medicine Institute, San José, Costa Rica

**Keywords:** erectile dysfunction, mesenchymal stem cells, shock wave therapy, hyperbaric oxygenation, regenerative medicine

## Abstract

**Background:**

Erectile dysfunction (ED) is a prevalent condition impacting men's quality of life and is often linked to cardiovascular and metabolic disorders. Conventional treatments like phosphodiesterase type 5 (PDE5) inhibitors could be ineffective for severe cases, indicating a need for innovative approaches. This study aimed to evaluate the efficacy and safety of a multimodal protocol combining low-intensity shockwave therapy (LiST), intrapenile and intravenous umbilical cord-mesenchymal stem cell (UC-MSCs) therapy, and hyperbaric oxygen therapy (HBOT) in men with ED. A retrospective pragmatic observational study was performed by reviewing medical records of 22 men treated at a private clinic in Costa Rica. Erectile function was measured using the Sexual Health Inventory for Men (SHIM) questionnaire before treatment and three months post-treatment.

**Results:**

The protocol significantly improved SHIM scores, with a mean increase of 3 points from baseline (*p* = 0.0017). No major adverse events were reported during follow-up.

**Conclusion:**

The multimodal protocol demonstrated a significant improvement in erectile function with a favorable safety profile, suggesting potential as a viable option for patients with ED. Further prospective randomized controlled trials are needed to validate these findings.

## Introduction

Erectile dysfunction (ED) is defined as the consistent inability to achieve or sustain an erection adequate for satisfactory sexual performance (1). It is a prevalent clinical condition, primarily affecting men over the age of 40 (2). The prevalence of ED is particularly high among middle-aged and elderly men, with projections estimating that the global number of affected individuals will reach 322 million by 2025 (3). Beyond its impact on sexual quality of life, ED is also recognized as an early indicator of cardiovascular disease, owing to its strong association with microvascular health (4).

Current treatments for ED primarily aim to alleviate symptoms rather than address the root causes of the condition. Phosphodiesterase type 5 (PDE5) inhibitors, such as sildenafil and tadalafil, are widely used and effective in many cases (5). However, their benefits are often temporary, and they may prove ineffective in patients with more severe underlying pathologies, such as diabetes-related ED or post-prostatectomy ED (6, 7). Furthermore, second and third-line therapies including vacuum erection devices, intraurethral suppositories, intracavernous injections, and penile implants—are associated with limitations such as side effects, high costs, and variable patient satisfaction (8, 9).In recent years, stem cell therapy has emerged as a promising treatment modality for ED. Stem cells, particularly mesenchymal stem cells (MSCs), have demonstrated the ability to differentiate into various cell types, promote angiogenesis, and exert paracrine effects that facilitate tissue repair and regeneration (10). Research has shown that MSCs derived from adipose tissue, bone marrow, and umbilical cords can improve erectile function in preclinical models through mechanisms involving the restoration of endothelial and smooth muscle function, as well as the modulation of inflammatory responses (11). A systematic review by Siregar et al. (2022) found that various types of stem cells, including placental matrix-derived stem cells, mesenchymal stem cell-derived exosomes, adipose-derived stem cells, bone marrow-derived mononuclear stem cells, and umbilical cord blood stem cells, have shown good efficacy and safety profiles in treating ED in humans (11).

Several clinical trials have demonstrated the potential benefits of stem cell therapy for ED. Bahk et al. (2010) reported that an intracavernous injection of human umbilical cord blood stem cells improved morning erections in 42.8% of diabetic patients with ED within one month and significantly increased the International Index of Erectile Function (IIEF) score at 11 months (12). Similarly, Levy et al. (2015) observed significant improvements in penile blood flow parameters and erectile function following the injection of placental matrix-derived mesenchymal stem cells in patients with Peyronie's disease and ED (13). In a subsequent study, Levy et al. (2016) found comparable results, reporting enhanced peak systolic velocity and overall erectile function (14). Haahr et al. (2016) demonstrated that a single intracavernous injection of autologous adipose-derived regenerative cells led to a significant increase in IIEF-5 scores in patients with ED following radical prostatectomy, with 47.0% of the patients showing marked improvement after 6 months (15). Yiou et al. (2017) further confirmed the safety and efficacy of bone marrow mononuclear cell injections in post-radical prostatectomy patients, with significant IIEF-5 score improvements at the 6-month follow-up (16).

Intravenous infusion of MSCs has also been demonstrated to enhance erectile function, primarily through paracrine mechanisms. These cells secrete neurotrophic factors, cytokines, and anti-inflammatory molecules that promote nerve regeneration, protect endothelial and smooth muscle tissue, and enhance hormonal balance (17–19). Thanh et al. utilized autologous adipose-derived MSCs, and 15 men with sexual dysfunction received intravenous infusions, with no occurrence of serious adverse events. Significant improvements were observed in erectile function, intercourse satisfaction, and overall satisfaction scores, along with a sustained increase in testosterone levels post-treatment (20).

In addition to stem cell therapy, alternative regenerative approaches, such as Low-Intensity Shockwave Therapy (LiST) and Hyperbaric Oxygen Therapy (HBOT), have demonstrated promising results in managing ED by addressing vascular insufficiencies and enhancing tissue health. LiST, is applied directly to the penis to induce microtraumas and stimulate blood vessel formation, thereby enhancing blood flow to the corpora cavernosa, which supports erections (21). This method encourages angiogenesis, neovascularization, improved penile blood flow, and endothelial function, offering an alternative for patients unresponsive to PDE5 inhibitors (22). Vardi et al. first demonstrated LiST's efficacy for ED in a 2010 pilot study, where 20 men with PDE5-sensitive ED saw a significant improvement in their IIEF-EF scores after six sessions over nine weeks, with effects lasting up to six months (23). Since then, numerous clinical trials, including 11 randomized controlled trials, have shown statistically significant improvements in IIEF scores in men with vasculogenic ED, with no serious adverse events reported (24, 25).

HBOT involves administering pure oxygen in a high-pressure chamber, designed to increase the partial pressure of oxygen in the blood and body tissues, which can aid in healing. This therapy may enhance erectile function by promoting vascularization, boosting endogenous nitric oxide production, and supporting penile tissue regeneration (26). Clinical studies suggest HBOT benefits erectile function. For instance, Yuan et al. conducted a randomized controlled trial with 24 patients, observing significantly higher IIEF scores in the treatment group after three months (27). Similarly, Hadanny et al. found an 88% improvement in erectile function in a prospective study of 30 patients with recent ED, while Sahin et al. and Sen et al. reported significant IIEF score improvements in cohort studies (28–30). None of these studies reported serious adverse events related to HBOT (27–30).

The combination of intrapenile injections of MSCs, intravenous MSC infusion, LiST, and HBOT represents a multimodal regenerative strategy that targets erectile dysfunction through complementary mechanisms. Intrapenile MSC injections deliver regenerative cells directly to the corpora cavernosa, enhancing local angiogenesis, modulating inflammation, and promoting smooth muscle regeneration, antifibrotic remodeling and neurotrophic support (31). When combined with systemic MSC infusion, the therapy broadens its impact by addressing underlying systemic or endothelial dysfunction and facilitating paracrine signaling that may enhance the homing and survival of locally administered cells (32). LiST further supports this approach by inducing controlled microtrauma, stimulating endogenous repair pathways, upregulating angiogenic factors such as VEGF, and increasing penile blood flow (33, 34). Also, induces controlled microtrauma to the corpus cavernosum, triggering a pro-repair environment that upregulates adhesion signals for MSC, NO pathways and angiogenic factor. Finally HBOT significantly raises tissue oxygenation levels, not only improving neovascularization but also supporting stem cell viability and function by creating a favorable oxidative environment (35). Together, these therapies act synergistically to improve vascularization, restore endothelial and smooth muscle integrity, and modulate the immune response, potentially leading to more robust and sustained improvements in erectile function than any single therapy alone.

This study explores the impact of a rapid multimodal treatment regimen, including LiST, intrapenile injections of MSCs, MSC intravenous infusion, and HBOT, on erectile function. The primary objective is to evaluate changes in erectile function using the Sexual Health Inventory for Men (SHIM) questionnaire following this integrative treatment. By examining the combined effects of these therapies, we aim to provide insights into the potential benefits of a multimodal strategy for ED, ultimately offering a more effective and comprehensive solution for patients.

## Methods

### Study design

This study was designed as a retrospective pragmatic observational study with a pre-post design based on medical records. The primary objective was to evaluate changes in the SHIM score and the incidence of adverse events among participants who underwent a multimodal treatment protocol for ED at a private clinic in Costa Rica between January 2022 and July 2023. The study was conducted at the Regenerative Medicine Institute (RMI), a medical facility specializing in regenerative medicine and cellular therapies, which is regulated by the Costa Rican Ministry of Health. The clinic has obtained the necessary authorization to offer expanded allogeneic mesenchymal stem cell treatments. The information gathered is derived exclusively from patient data collected within a clinical context.

### Participants

The study population comprised 22 male participants with a clinical diagnosis of erectile ED who underwent the multimodal treatment protocol at RMI with an interest to undergoing a regenerative medicine approach. Participants were included if they met the following criteria: Firstly, a diagnosis of ED according to the SHIM scale was required, with a score of <21 (36). Secondly, participants had to be aged 18 years or older. Thirdly, treatment had to have been received at RMI between January 2022 and July 2023. Finally, complete SHIM scores had to be documented at both baseline and at the 3 months follow-up calls. Participants were excluded if they had a history of radical prostatectomy as this population typically has severe neurogenic ED and was expected to respond differently to regenerative approaches, penile fracture, coagulopathies, cancer (either history or active), were over 75 years old, had chronic decompensated diseases, or presented contraindications for sedation or hyperbaric oxygen therapy.

### Collection and processing of UC-MSCs

The collection of UC-MSCs followed standardized procedures designed to ensure both the quality and safety of the harvested cells. In compliance with relevant regulations, informed consent was obtained from umbilical cord donors at the time of cesarean delivery, allowing for the donation of biological material. A thorough review of the donor mother's medical history was performed to rule out hereditary conditions and any history of oncological disease. Additionally, the donor's health was assessed by the attending physician before delivery. Days previously to the cesarean section, maternal blood samples were collected to screen for infectious diseases. On the scheduled day of the cesarean section, a umbilical cord blood sample was collected for serological screening to detect transmissible infections. These test results were directly linked to the corresponding umbilical cord batch. If any sample tested positive for an infectious pathogen, the associated umbilical cord was excluded from further processing. Only those cords that met all established safety and screening requirements were deemed suitable for use.

Immediately after collection, the umbilical cords were transported to a laminar flow chamber for processing under sterile conditions. The blood vessels were dissected and removed, and the remaining tissue was thoroughly washed to eliminate any residual blood. Small sections were then extracted and tested for *Mycoplasma sp*. to ensure the absence of contamination. Following this step, the tissue segments were placed in culture flasks containing alpha MEM medium supplemented with 10% fetal bovine serum and incubated for 15 days. Throughout this period, the culture medium was refreshed every 2–3 days until the cells reached confluency. Once confluence was attained, the cells underwent *in vitro* expansion up to passage five. At this stage, a sample was collected for quality assessment, including re-screening for Mycoplasma sp. and evaluation of multipotency by inducing differentiation into chondrogenic, osteogenic, and adipogenic lineages.

Additionally, flow cytometry was employed to analyze surface marker expression, specifically assessing positive markers such as CD90, CD73, CD105, and CD44, while negative markers included CD34, CD19, CD45, and CD11b. Microbiological cultures were conducted to confirm the absence of bacterial and fungal contamination, and an RT-PCR test was performed to detect Mycoplasma sp. Genetic stability was further verified through karyotype analysis. Cells that met all quality control standards were cryopreserved using a specialized cryoprotectant and stored in vials at −80°C.

Before application, thawed cells were evaluated using trypan blue staining to determine the total cell count and assess the percentage of viable cells.

The laboratory is regulated by the Costa Rican Ministry of Health and has all the required permissions to harvest and expand allogenic mesenchymal stem cells as a tissue bank. It is also certified by ISO 7 and operates according to GMP standards.

### Multimodal protocol

Prior to the procedure, all patients received a standardized premedication regimen consisting of intravenous dexamethasone 4 mg, oral famotidine 40 mg, and oral loratadine 10 mg. The multimodal protocol was carried out in four phases, with the first three performed sequentially during a single procedural session under intravenous sedation and continuous monitoring. The last phase includes four sessions of hyperbaric oxygen therapy, which are administered over two to three days.

The session began with low-intensity shockwave therapy (LiST), administered using the F-SW Ultra device (Storz Medical), delivering between 5,000 and 6,500 shockwaves at an energy flux density of 0.5 mJ/mm² and a frequency of 6 Hz. These settings targeted the penile tissue with a higher total pulse count intended to induce microinjury in a single session, rather than through cumulative multiple sessions. Immediately afterward, a single intravenous infusion of 50 million umbilical cord-derived mesenchymal stem cells (UC-MSCs), diluted in 500 ml of Ringer's lactate, was administered over the course of one hour, representing a reasonable systemic dose for immunomodulation and vascular milieu enrichment. Following the infusion, intrapenile injections of UC-MSCs were performed under high-resolution ultrasound guidance. Within the dosing range reported in published trials, a total dose of approximately 25 million cells, suspended in 6 ml of Ringer's lactate, was injected into six anatomical sites along the corpora cavernosa, three injections per side, with 1 ml per site. These doses align with the range of concentrations employed in other studies that have yielded encouraging outcomes.

Within the subsequent 2–3 days, depending on hyperbaric center availability, participants underwent four sessions of HBOT to potentiate UC-MSC survival and function. Each session was conducted in a Sechrist monoplace chamber at 2.8 atmospheres for 60 min. These settings are consistent with the results previously reported in the scientific literature, which contribute to preserving the viability of stem cells.

### Outcome measurement

The primary outcome of this study was the improvement in erectile function, assessed using the Sexual Health Inventory for Men (SHIM) questionnaire. SHIM scores were collected at baseline and at a follow-up visit three months post-treatment. This validated tool quantifies erectile function on a scale from 1–25, where lower scores indicate more severe erectile dysfunction. The change in SHIM score from baseline to follow-up served as the primary metric of treatment efficacy. As a minimal clinical importance change (MCID) the value of ≥4 will be used according to what is stated by Rosen et al. on their article (37).

Secondary outcomes included the assessment of any adverse events and the rate of reintervention. Adverse events were recorded throughout the treatment period and 3 months follow-up phase, focusing on any immediate complications during treatment sessions and at each follow-up point. Incidents necessitating additional treatment, categorized as reinterventions, were documented along with the underlying reasons.

### Data collection

Systematic data collection was carried out using the medical records of participants who underwent the multimodal protocol. The extracted data included clinical, sexual, and sociodemographic characteristics; SHIM scores at baseline and follow-up visits; adverse events and complications; and instances of reintervention along with their respective causes. All data were anonymized and stored in a secured database accessible exclusively to the research team.

### Statistical analysis

All statistical analyses were conducted using Stata software (version 18.5; StataCorp LLC, College Station, TX). Before performing hypothesis testing, the distribution of SHIM scores was evaluated using the Shapiro–Wilk test, which indicated that the data followed a normal distribution (*p* > 0.05). Descriptive statistics were applied to summarize clinical, sexual, and sociodemographic characteristics. Continuous variables were expressed as means, median and standard deviations (SD), while categorical variables were reported as frequencies and percentages. To assess changes in SHIM scores between baseline and 3 months post-treatment, a paired *t*-test was utilized, as it was appropriate for the matched observations. The results were reported as mean differences with 95% confidence intervals (CI), and a two-tailed significance level of 5% was used to define statistical significance.

Finally, a linear regression model was used to evaluate the relationship between the SHIM score, treated as a continuous outcome, and several predictors: time (baseline vs. 3 months), testosterone use (binary: yes/no), and presence of comorbidities were all managed as binary variables. ED severity functioned as a categorical variable according to the SHIM's interpretation; and age, which was treated as a continuous variable.

The analysis was conducted in three stages. First, univariate models were estimated for each predictor separately to quantify their crude associations with SHIM. Second, the univariate model containing only time was selected as the crude representation of the time-to-outcome relationship. Finally, a multivariate adjusted model was constructed including all predictors simultaneously, allowing the estimation of the independent effect of each variable while accounting for the others.

This approach allowed the test for potential confounding. Collinearity was carefully assessed by comparing crude and adjusted models.

To explore potential heterogeneity of treatment effect, subgroup analyses were also performed by stratifying patients according to testosterone use (users vs. non-users). In addition, interaction testing was performed by including a multiplicative term in the regression model to evaluate whether testosterone use modified the effect of time on SHIM outcomes. All models were estimated using robust standard errors clustered by participant ID to account for repeated measures within individuals. Model performance was evaluated using the coefficient of determination (R²) to assess explanatory power and the root mean square error (RMSE) to measure precision. Results are reported as regression coefficients (β) with 95% confidence intervals and *p*-values.

## Results

The study included 22 male participants diagnosed with ED who received the multimodal treatment protocol at the RMI in Costa Rica between January 2022 and July 2023. Table 1 presents a detailed summary of the participants' demographic characteristics, clinical profiles, and lifestyle factors, which are essential for understanding the context and potential impact of the treatment outcomes.

**Table 1:** Baseline demographic and clinical characteristics of the study population.

Variable	Summary measures	*N* (%)
Age	Mean	63.0 (SD 8.21)
Marital status	Married	14 (63.64%)
	Divorced	4 (18.18%)
	Single	3 (13.64%)
	Not available	1 (4.55%)
Weight	Mean	88.49 kg (SD 12.01)
Height	Mean	179.23 cm (SD 7.56)
Body Mass Index	Mean	26.57 (SD 3.17)
Comorbidities	Dyslipidemia	7 (31.8%)
	Cardiovascular	7 (31.8%)
	None	6 (27.3%)
	History of cancer	2 (9.0%)
	Gout	2 (9.0%)
	Diabetes mellitus	2 (9.0%)
	Respiratory	1 (4.5%)
	Musculoskeletal	1 (4.5%)
	Hypothyroidism	1 (4.5%)
Medications	None	6 (32%)
	Antihypertensives	7 (27.27%)
	Multivitamin Supplements	4 (18.18%)
	Statins	7 (27.27%)
	Antidiabetics	2 (9.0%)
	Anti-inflammatories and analgesics	1 (4.5%)
	Others	1 (4.5%)
	Levothyroxine	1 (4.5%)
Use of ED medications	Yes	9 (40.9%)
	No	13 (59.1%)
Testosterone supplementation	Yes	7 (31.8%)
	No	9 (40.9%)
	Not available	6 (27.3%)
Physical activity level (days per week)	0	8 (36.36%)
	4	5 (22.73%)
	5	5 (22.73%)
	6	1 (4.55%)
	7	3 (13.64%)
Smoking	Active	5 (22.73%)
	Inactive	4 (18.18%)
	Non smokers	10 (45.45%)
	Not available	3 (13.64%)
Alcohol	Active	14 (63.64%)
	No history of alcohol consumption	5 (22.73%)
	Not available	3 (13.64%)
Recreational drug use	Active	2 (9.09%)
	No history of recreational drug use	13 (59.09%)
	Not available	7 (31.82%)
Previous surgeries	Orthopedic	13 (59.9%)
	General	4 (18.18%)
	Cardiovascular	2 (9.0%)
	ENT	2 (9.0%)
	Urology	1 (4.55%)
	Ophthalmic	1 (4.55%)
	Denies	5 (22.73%)
Basal Testosterone Level	Mean	750.96 ng/dl (SD 484.59)

### Baseline SHIM scores

The mean baseline SHIM score for the participants was 13.59 (SD = 5.51), indicating varying levels of ED severity within the study population. Some participants experienced more pronounced erectile difficulties than others. The SHIM scores served as a quantitative measure to assess the effectiveness of the multimodal treatment by comparing scores before and after the intervention.

### Grade of erectile dysfunction

**Figure 1:**
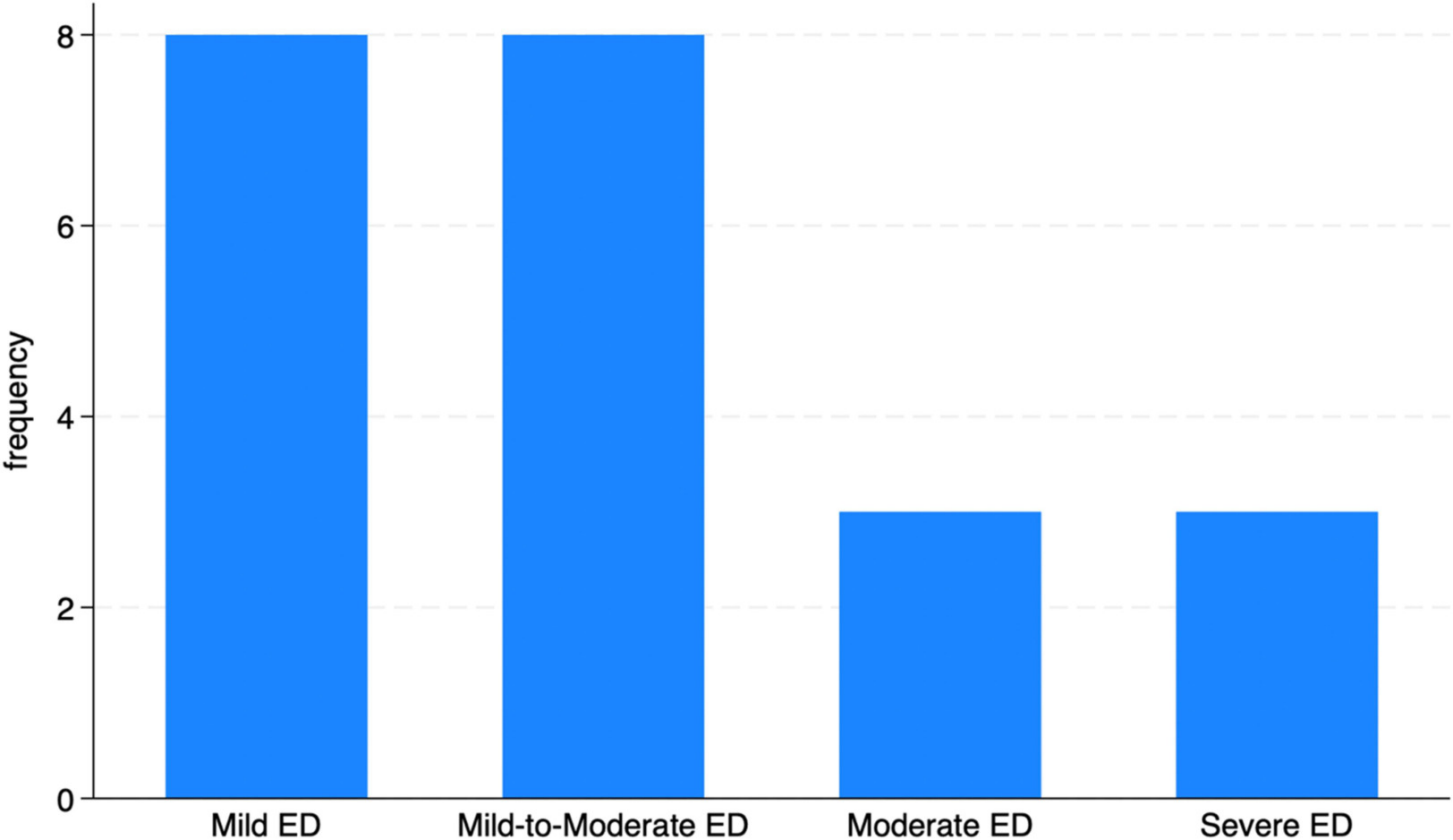
Frequency of ED at baseline visit according to the SHIM scale.

Figure 1 illustrates the distribution of ED severity at the first visit. At baseline, the distribution of ED severity among participants showed that most were in the mild to moderate range. Determining baseline SHIM scores and ED grades was essential for evaluating the efficacy of the treatment protocol, as it allowed for a comparison of erectile function before and after the intervention.

### SHIM score and treatment outcomes

**Figure 2:**
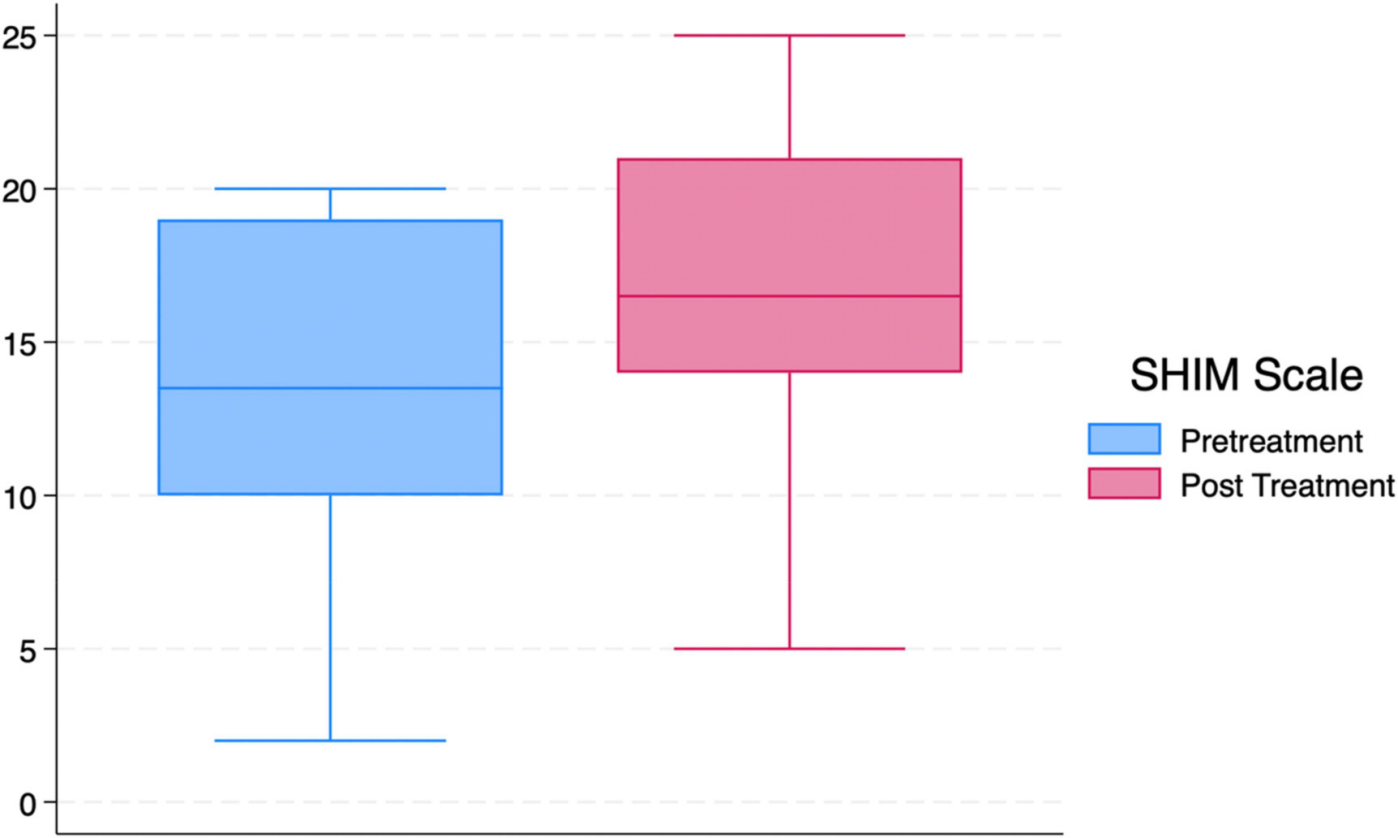
Baseline and 3-month follow up SHIM scale results.

A paired *t*-test was performed to assess changes in erectile function, as measured by the SHIM score, from baseline to 3 months post-treatment. The mean SHIM score at baseline was 13.59 (SD = 5.51), increasing to 16.59 (SD = 5.94) (*p* = 0.0017) at 3 months as shown in Figure 2, reflecting an average improvement of 3.00 points (95% CI: 1.26–4.74). Using the previously mention MCID on the SHIM scale, 40.9% (*n* = 9) of the participants had this improvement.

**Figure 3:**
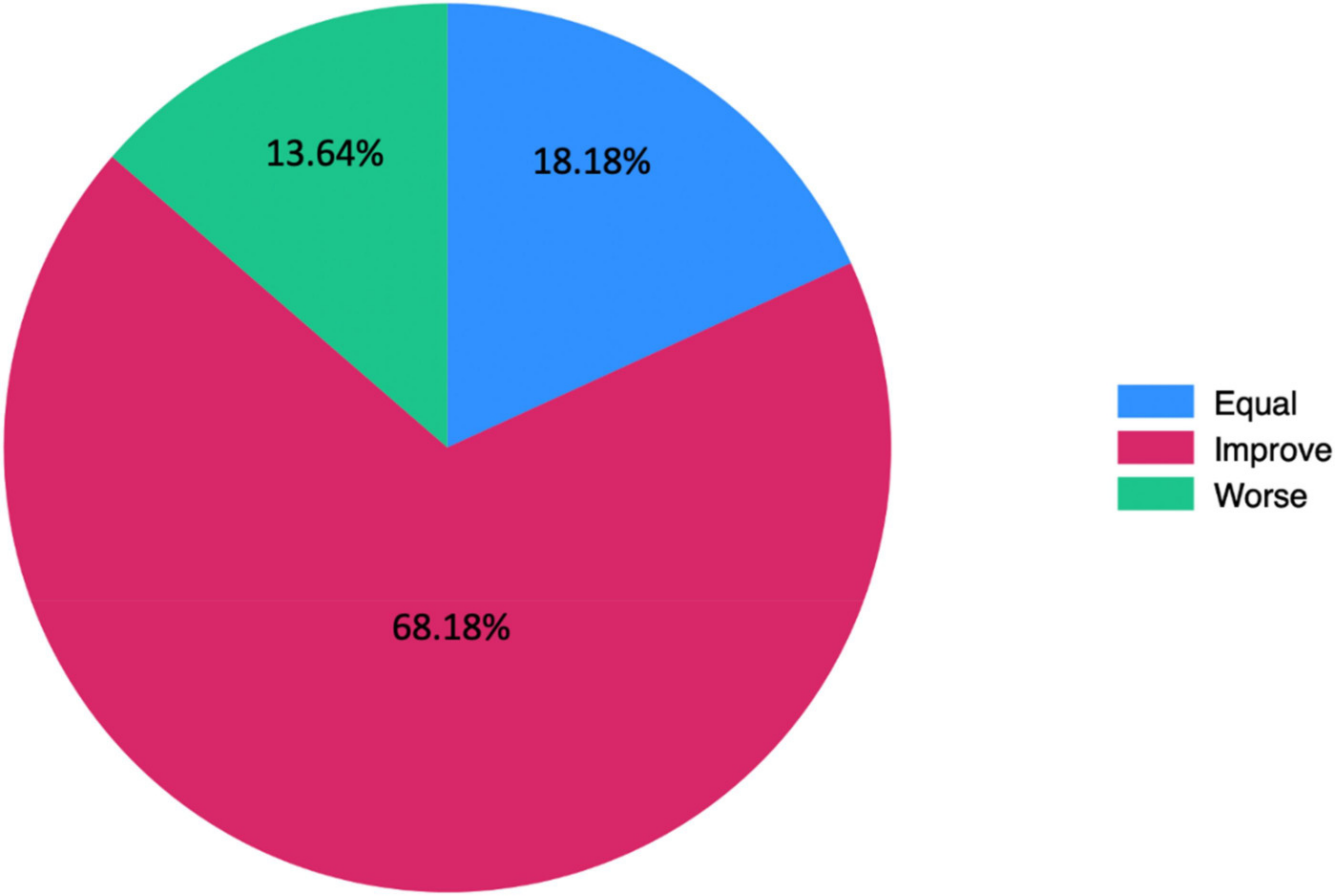
Percentage of participants reporting improvement, no change, or worsening in erectile function severity post-treatment.

Treatment outcomes are illustrated in Figure 3. Of those patients who reported some degree of improvement, two thirds showed a shift to a lower grade of erectile dysfunction, while the remaining experienced improvement without a change in severity grade. Two participants (9.09%) required reintervention to enhance outcomes, while the other 20 patients (90.91%) did not require additional interventions because they achieved a satisfactory results.

### Regression models

In Table 2, the results of both unadjusted and adjusted model can be appreciated. In the univariate analysis, follow-up at 3 months was associated with a statistically significant improvement in SHIM scores, with an average increase of 3.63 points (95% CI: 1.18–6.07; *p* = 0.006). Higher baseline ED severity was consistently associated with lower SHIM scores. In contrast, comorbidities were not significantly associated with SHIM in univariate models.

**Table 2:** Association between predictors and SHIM score in univariate and adjusted models.

Predictor	Univariate β (95% CI)	*p*-value	Adjusted β (95% CI)	*p*-value
Time (3 months vs. baseline)	3.00 (1.24, 4.76)	0.002	3.63 (1.18, 6.07)	0.006
Age (years)	−0.35 (−0.57, −0.12)	0.004	−0.01 (−0.24, 0.23)	0.949
Testosterone use	−8.05 (−11.88, −4.22)	<0.001	−1.92 (−5.17, 1.33)	0.227
Severity of ED				
Mild–moderate	−4.74 (−7.35, −2.13)	0.001	−3.53 (−6.19, −0.86)	0.013
Moderate	−6.04 (−9.35, −2.72)	0.001	−3.61 (−6.35, −0.86)	0.014
Severe	−13.19 (−16.89, −9.49)	<0.001	−10.50 (−16.03, −4.97)	0.001
Comorbidity (app_binary)	−3.54 (−7.68, 0.60)	0.090	−1.54 (−4.82, 1.74)	0.333

When adjusting for all predictors in the multivariate model, the association between 3-month follow-up and SHIM score remained significant, with an adjusted mean increase of 3.63 points (95% CI: 1.18–6.07; *p* = 0.006).

### Subgroup analysis and interaction testing

When stratifying by testosterone use, both users and non-users demonstrated improvements in SHIM scores at 3 months compared to baseline. Participants who did not use testosterone showed an average increase of 3.8 points, which was borderline significant (*p* = 0.058). Those who used testosterone improved by 3.4 points, and this effect reached statistical significance (*p* = 0.014).

To formally test whether the effect of time differed by testosterone use, an interaction term between time and testosterone use was included in the regression model. The interaction term was small (*β* = −0.35, *p* = 0.877) and not statistically significant, indicating no evidence that testosterone use modified the treatment effect. Margins analysis confirmed that the direction and magnitude of improvement were consistent across both groups.

A complete summary of regression coefficients, confidence intervals, and *p*-values for both the univariate and adjusted models is presented in Table 3.

**Table 3:** Subgroup analysis of change in SHIM scores by testosterone use.

Testosterone use	Change in SHIM (β)	95% CI	*p*-value
No testosterone	3.78	−0.15 to 7.71	0.058
Uses testosterone	3.43	0.80 to 6.05	0.014

### During procedure and immediate post-treatment complications

No complications occurred during the administration of the multimodal protocol. All procedures, comprising LiST, intrapenile MSC injections, intravenous MSC infusions, and HBOT, were performed without any immediate post treatment complications, indicating a high level of procedural safety for the interventions included in the protocol.

### Follow-up adverse events

Participants were closely monitored for adverse events during the first month post-treatment and at subsequent 3-month follow-up visits. Notably, no adverse events were reported by any participants during the three months, suggesting that the treatment protocol was well-tolerated.

## Discussion

This study evaluated the effects of a regenerative multimodal protocol combining LiST, intrapenile and intravenous infusion of UC-MSCs, and HBOT in men with ED. The findings showed a mean improvement of 3.0 SHIM points after three months, increasing to 3.63 points after adjusting for baseline variables. These results suggest that the multimodal approach offers potential functional benefits, though the clinical impact varies among individuals. Only 40.9% of participants reached the MCID of ≥4 points, as defined by Rosen et al. (37).

The results of this study align with the findings of previous research on regenerative therapies for ED, particularly those stem cell–based interventions. According to the findings of Levy et al. and Haahr et al., improvements in SHIM and IIEF scores have been reported following intracavernous administration of MSCs (13–15). Unlike those single-modality trials, our approach integrated complementary therapies with the objective of targeting multiple pathophysiological pathways.

LiST has been demonstrated to promote neovascularization and enhance penile hemodynamics, particularly in patients with vasculogenic ED (38). A series of randomized controlled trials have documented significant improvements in IIEF scores without major adverse events, supporting its safety and efficacy (23, 39). HBOT has shown positive results in select clinical studies, with evidence suggesting improvements in tissue oxygenation, stimulation of angiogenesis, and support for nitric oxide–mediated vasodilation (28, 29).

A key finding of this study is the absence of intraoperative or postoperative complications or adverse events during the three-month follow-up period. This finding was particularly relevant given that the multimodal protocol involved multiple interventions performed sequentially, including systemic and local cell delivery under sedation. Previous clinical trials of MSC therapy, LiST, and HBOT have similarly reported minimal safety concerns, but data on combined use remains scarce (23, 38, 40, 41). Our findings suggest that the integrative approach does not increase procedural risk, providing evidence of its short-term safety.

The regression analysis provided additional findings; the variable time captures the effect of exposure to the multimodal regenerative protocol. The crude model suggested an average improvement of 3.0 SHIM points at 3 months, which increased to 3.63 points after adjustment. This strengthening of the coefficient implies that the effect of protocol exposure was somewhat attenuated in the crude estimate, likely due to individual variability. By accounting for baseline characteristics, the adjusted model more clearly isolates the independent contribution of the multimodal intervention to functional improvement.

The regression analysis showed that baseline ED severity was the strongest negative predictor of post-treatment SHIM, consistent with the concept that advanced neurovascular and structural damage is less reversible, even with regenerative strategies (42). Age was associated with lower SHIM scores in univariate analysis but lost significance after adjustment, suggesting possible confounding by baseline severity. Testosterone use and comorbidities were not independently associated with SHIM outcomes after adjustment.

The subgroup analysis revealed statistically significant improvements in SHIM scores among participants using testosterone and borderline improvements among those not using testosterone. However, interaction testing confirmed that the effect of time was consistent across both groups, with no evidence of effect modification. This suggests that the protocol benefits men regardless of testosterone use, and that the observed subgroup differences likely reflect sample size limitations rather than true heterogeneity of effect.

From a clinical perspective, these results support the feasibility and short-term safety of a multimodal regenerative protocol in a heterogeneous ED population. The absence of major complications is worth noting, especially given the procedural complexity. However, the small proportion of patients who achieve the MCID underscores the need for a more refined patient selection process. Individuals with milder disease may derive greater benefit, while those with severe ED or multiple comorbidities might require adjunctive or repeated interventions.

Given the substantial cost and logistical requirements of this protocol, its integration into routine practice would necessitate evidence from larger, controlled trials demonstrating both superior efficacy over conventional treatments and favorable cost-effectiveness.

It is important to acknowledge the methodological limitations of the study. The retrospective design of the study, in conjunction with the absence of a control group, limits the ability to make causal inferences. This suggests the possibility that placebo effects or regression to the mean may have contributed to the observed improvements. The limited sample size and the heterogeneity in comorbidities and the use of concurrent therapies resulted in reduced statistical power and limited the execution of meaningful subgroup analyses. Additionally, the three-month follow-up period is insufficient for evaluating the long-term durability of treatment effects. Finally, while the safety profile was favorable, the absence of objective penile hemodynamic measures, such as Doppler ultrasound, limits the ability to make a correlation between functional gains and physiological changes.

It is recommended that future research prioritizes the execution of randomized controlled trials with larger and more homogeneous cohorts to validate these preliminary findings. It is imperative that future studies investigate the optimal dosing strategies for UC-MSCs. These studies should also examine the ideal timing and frequency of LiST and HBOT sessions. Additionally, research is needed to determine the long-term maintenance of clinical improvements. The incorporation of objective vascular and neurophysiological endpoints would serve to enhance the mechanistic understanding of the subject and guide the refinement of the protocol.

## Conclusion

This study shows the results of a multimodal approach combining low-intensity shockwave therapy, intrapenile and intravenous MSC therapy, and HBOT. Participants showed an increase in SHIM scores that is statistically significant and close to the MCID, suggesting that the integrative protocol may offer a therapeutic option. Also, the absence of adverse events gives a good safety profile for this intervention.

The absence of intraoperative and postoperative complications further supports the safety profile of this treatment combination. Although these results are promising, the study's retrospective design and the lack of a control group limit the ability to establish causality. Future randomized controlled trials with larger sample sizes and longer follow-up periods are needed to confirm these findings and to determine the optimal therapeutic combination and frequency of treatments.

In summary, the multimodal protocol offers an effective and safe treatment option for ED, addressing multiple pathways involved in erectile function restoration. This study provides a foundation for further research to refine and validate the approach, ultimately offering hope for improved outcomes in patients with complex or treatment-resistant ED.

## Data Availability

The raw data supporting the conclusions of this article will be made available by the authors, without undue reservation.
