# Supplementary material for: Enhancing penile function: the impact of a regenerative multimodal protocol on erectile dysfunction
Source: Front Reprod Health. 2025 Sep 29;7:1601354. doi: 10.3389/frph.2025.1601354 (PMC12515801; doi:10.3389/frph.2025.1601354)
Supplement: Supplementary file 1 [file Supplementaryfile1.docx]

**Univariate models:**

1.
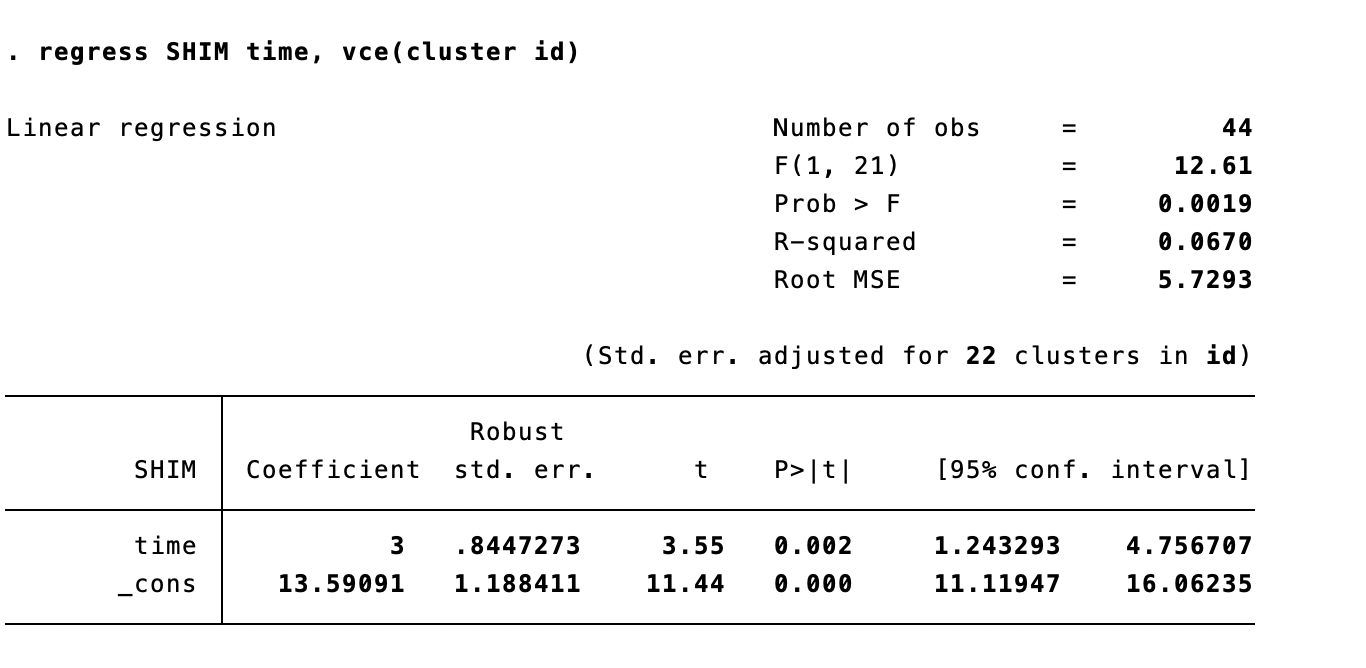
**Time (also Crude Model)**
2. **Severity of Erectile Dysfunction:**


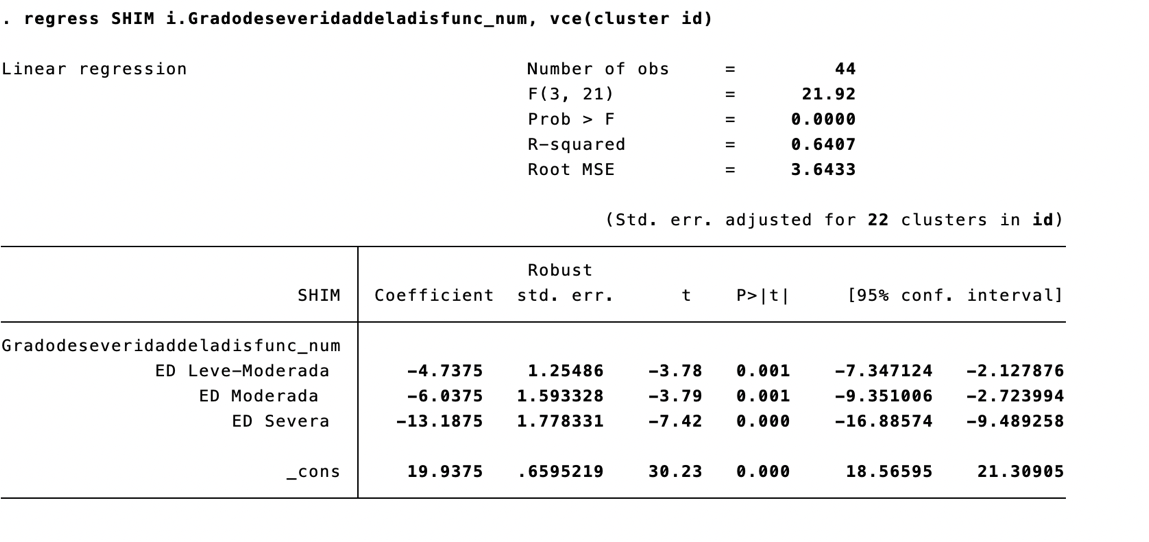


1.
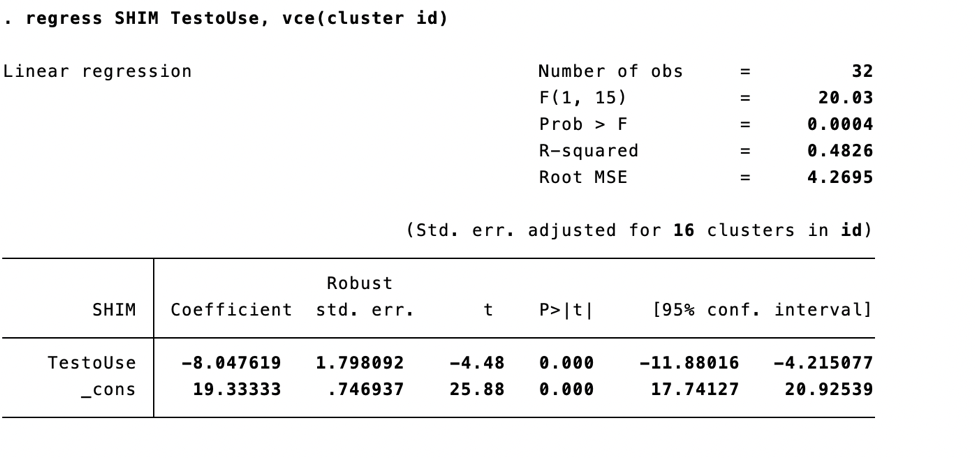
**Testosterone Use:**
2.
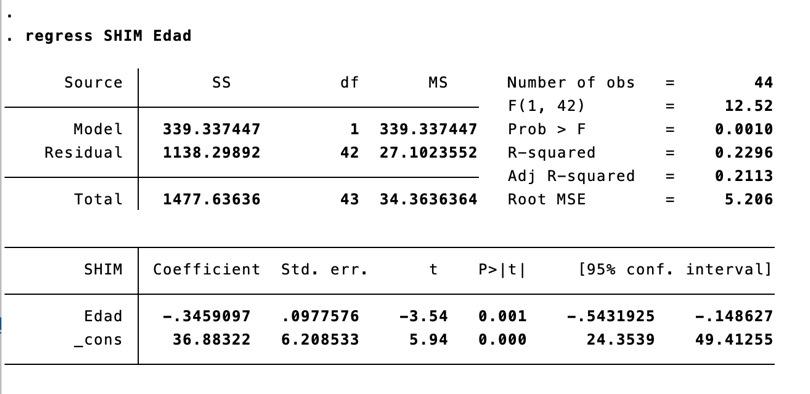
**Age**
3.
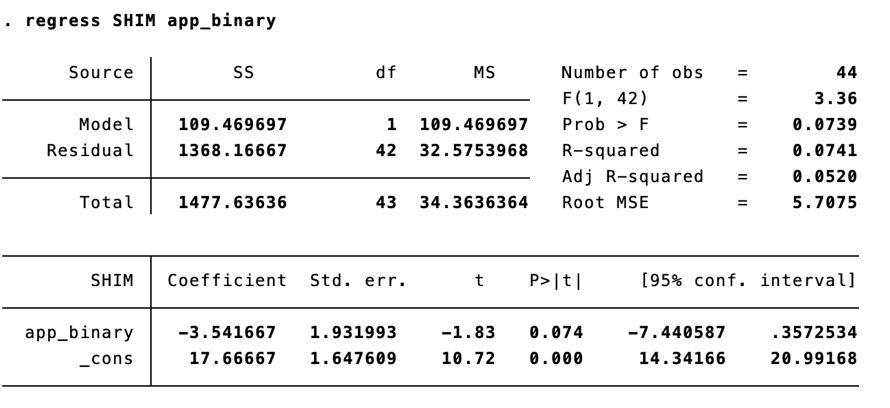
**Comorbidities**

**Regressions models:**

1. **Unadjusted association between time and SHIM score**


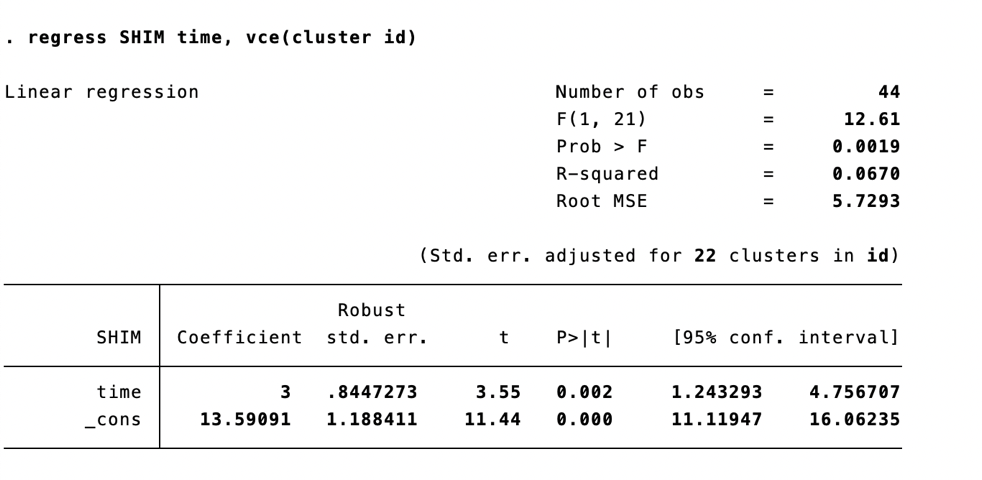


1.
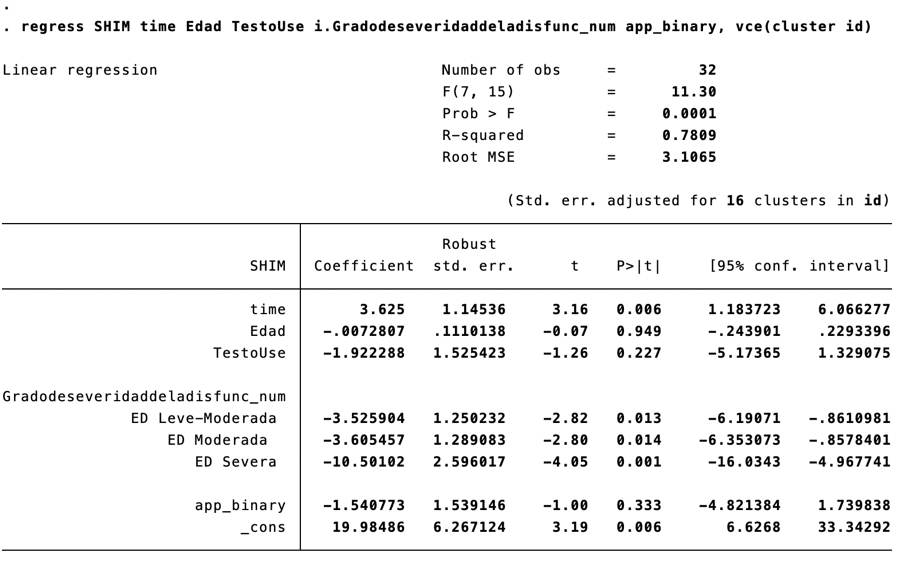
**Multivariate regression (representing the adjusted model)**
2.
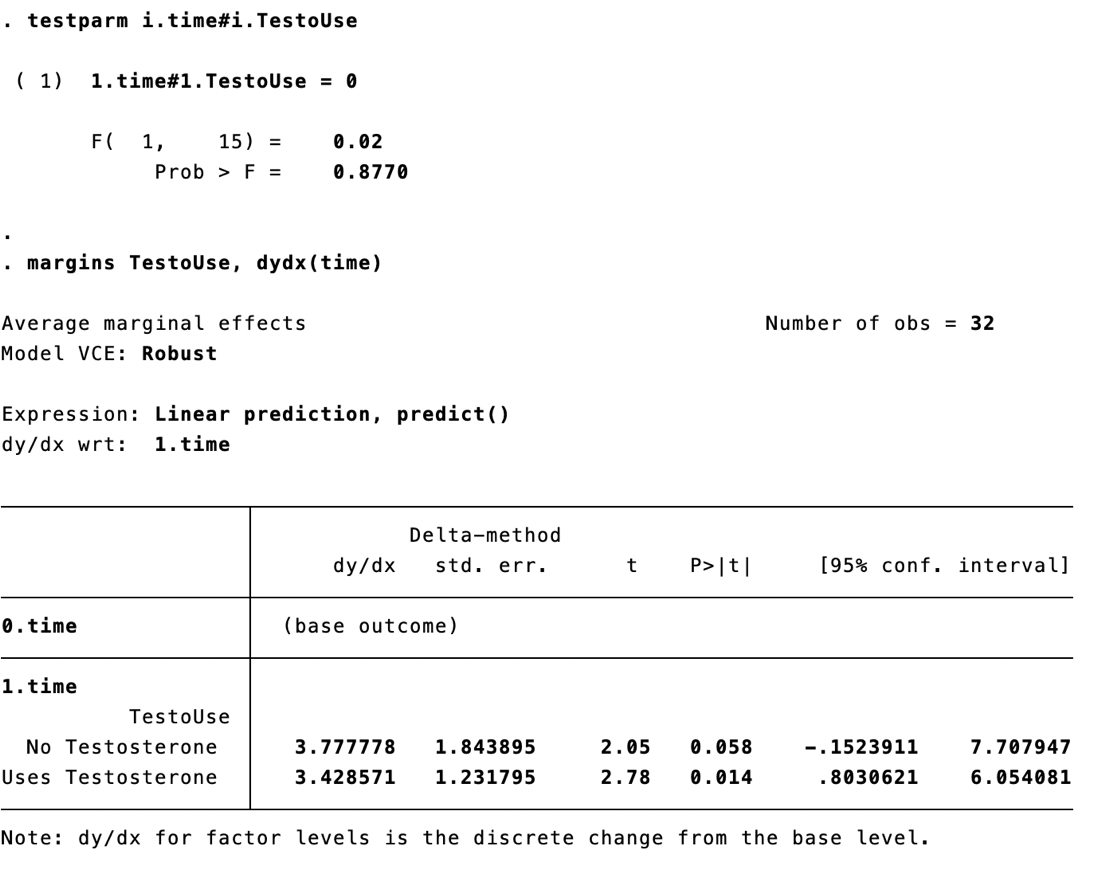
**Subgroup analysis with binary testosterone use variable**
3. **
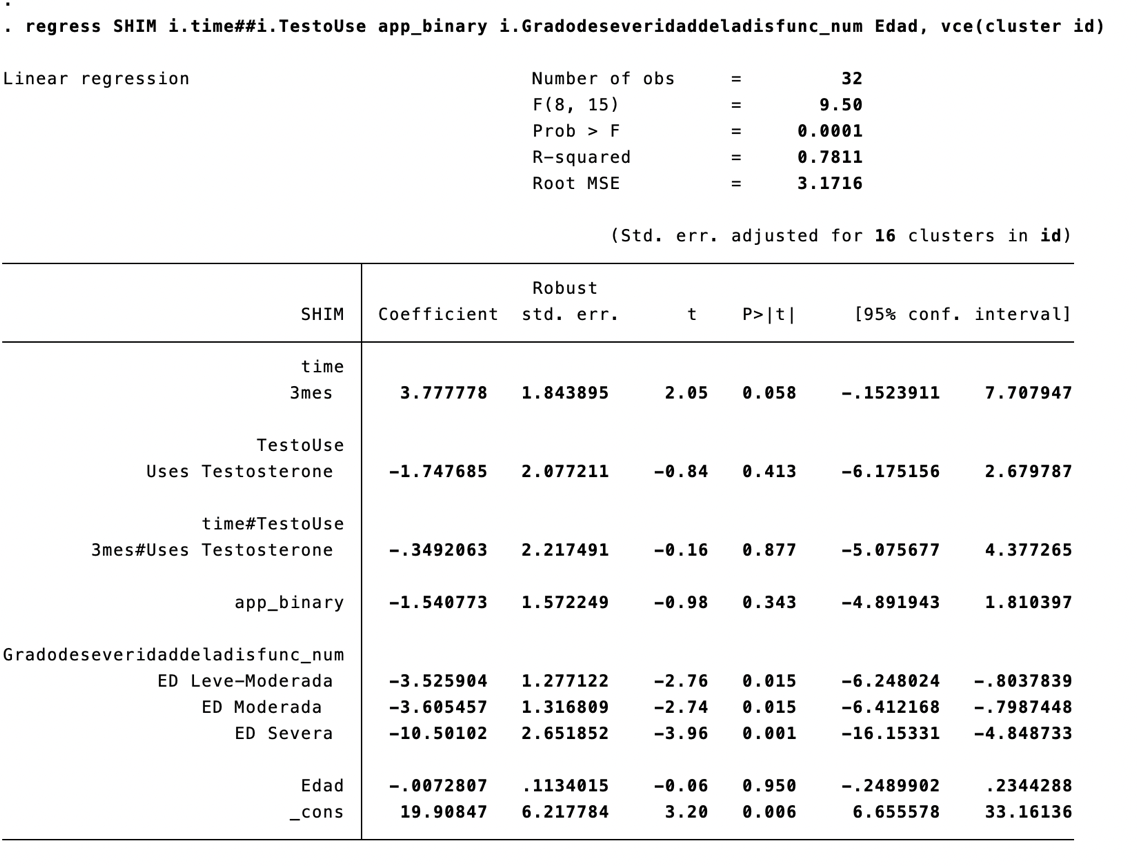
Interaction term for time and binary testosterone use variable**
